# Delivery of Cancer Therapeutics Using Nanotechnology

**DOI:** 10.3390/pharmaceutics5020294

**Published:** 2013-05-15

**Authors:** Eun-Kyung Lim, Eunji Jang, Kwangyeol Lee, Seungjoo Haam, Yong-Min Huh

**Affiliations:** 1 Department of Radiology, Yonsei University, 50 Yonsei-ro, Seodaemun-gu, Seoul 120-752, Korea; E-Mail: eklim@yuhs.ac; 2 Department of Chemical and Biomolecular Engineering, Yonsei University, 50 Yonsei-ro, Seodaemun-gu, Seoul 120-749, Korea; E-Mail: ozz@yonsei.ac.kr; 3 Department of Chemistry, Korea University, 145 Anam-ro, Dongdaemun-gu, Seoul 136-701, Korea; E-Mail: kylee1@korea.ac.kr

**Keywords:** nanoparticles, nanotechnology, drug delivery, cancer, theranostic nanoparticles

## Abstract

Nanoparticles have been investigated as drug carriers, because they provide a great opportunity due to their advantageous features: (i) various formulations using organic/inorganic materials, (ii) easy modification of targeting molecules, drugs or other molecules on them, (iii) effective delivery to target sites, resulting in high therapeutic efficacy and (iv) controlling drug release by external/internal stimuli. Because of these features, therapeutic efficacy can be improved and unwanted side effects can be reduced. Theranostic nanoparticles have been developed by incorporating imaging agents in drug carriers as all-in-one system, which makes it possible to diagnose and treat cancer by monitoring drug delivery behavior simultaneously. Recently, stimuli-responsive, activatable nanomaterials are being applied that are capable of producing chemical or physical changes by external stimuli. By using these nanoparticles, multiple tasks can be carried out simultaneously, e.g., early and accurate diagnosis, efficient cataloguing of patient groups of personalized therapy and real-time monitoring of disease progress. In this paper, we describe various types of nanoparticles for drug delivery systems, as well as theranostic systems.

## 1. Introduction

Since polyalkylcyanoacrylate nanoparticles attached with anticancer drugs were described in the late 1970s, nanotechnology has been an emerging field in drug-delivery research, which aims to design drug carriers that deliver drugs more precisely to tumor cells and maintain them at a therapeutic concentration over a long period [1,2]. Nanocarriers for drug delivery are referred to as drug-incorporated macromolecular soft matrixes or inorganic solid, colloidal particles within a size of 10–1000 nm, which is similar to that of biological macromolecules, such as proteins and DNA [3]. Because nanocarriers can be precisely fabricated with proper size, shape, surface charge, stability and various other characteristics for *in vivo* applications, nanocarrier-based drug delivery has been most extensively explored [4]. To further enhance their functionality, additional techniques, such as surface modifications, e.g., introducing a targeting moiety, are basically included in these approaches. For effective anticancer treatment, the drug must penetrate the tissue efficiently. Thus, studies suggested strategies to improve drug penetration through tumor tissue to enhance the therapeutic index by considering several delivery system on the basis of their abilities to penetrate tissue with an understanding of the physiological aspects of cancer [5]. For example, the vascular architecture around cancer is known to be poorly organized with reduced vascular density, irregular blood flow and compression of blood and lymphatic vessels by cancer cells [5]. In addition, numerous discoveries of biomolecular markers that are specifically expressed in cancer have been reported, which improved the understanding of cancer and resulted in targeted cancer therapies [6]. On the basis of these aspects, clinical approaches with nanotechnology have proven that drug delivery systems can show enhanced efficacy, while simultaneously reducing side effects, owing to properties, such as improved targeted delivery to tumors and active cellular uptake [7]. This review introduces drug delivery systems using nanotechnology, including general preparation of drug nanocarriers, strategies for their accumulation into cancer cells and recent advances in cancer therapy.

## 2. Common Nanostructures for Drug Carriers: Their Structures and Drug-Loading Principles

### 2.1. Supramolecular Organic Architectures

#### 2.1.1. Polymeric Micelles

**Figure 1:**
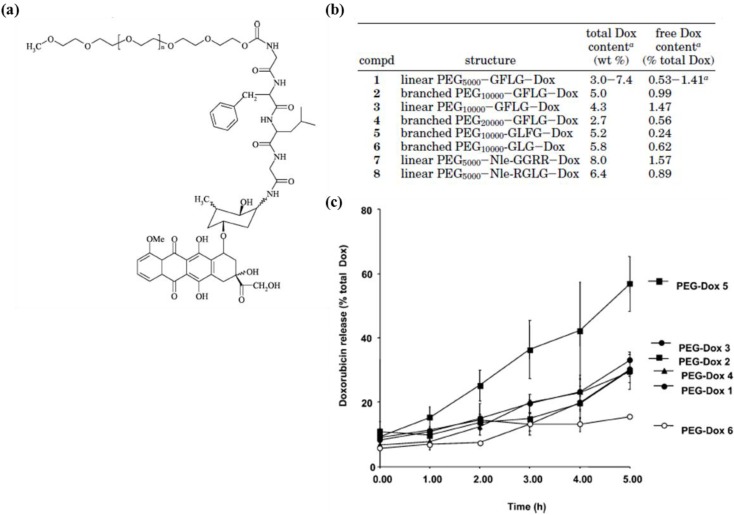
(**a**) Structure and (**b**) composition of the poly(ethylene glycol) (PEG)-peptide-doxorubicin (DOX) conjugates. Release of DOX from PEG-peptide-DOX conjugates during incubation with isolated rat liver lysosomal enzymes (tritosomes). Reproduced from [9] with permission of [American Chemical Society Publications].

A polymeric micelle structure, consisting of a hydrophilic shell and a hydrophobic core, is commonly adapted in drug delivery systems, due to its tunable size and surface functionality, high monodispersity and excellent stability [8]. The polymers used for micelles range from simple natural polymers to exquisite synthetic copolymers, which have a generally amphiphilic characteristic. With regard to the drug-loading principles using this amphiphilic micelle structure, there are two typical routes: drug conjugation and drug encapsulation. Drug conjugation utilizes a non-water soluble drug as a hydrophobic core of the micelle, which is conjugated to the hydrophilic polymer backbone. For drug release, biodegradable chemical linkers are usually selected for conjugating the drug to the main chain. For example, Duncan *et al.* studied poly(ethylene glycol) (PEG)-doxorubicin (DOX) conjugates with peptide linkers. Their study covered several factors for drug delivery, e.g., drug release profiles, *in vitro* cytotoxicity and biodistribution, with regard to PEG-DOX polymers of linear or branched architecture (molecular weight 5000–20,000 g/mol) and with different peptidyl linkers (GFLG, GLFG, GLG, GGRR and RGLG) (Figure 1) [9]. The second route is drug encapsulation, *i.e.*, drug-loading micelles are formed by emulsion of the drug with ready-made amphiphilic copolymers. In this case, drugs are physically entrapped into the hydrophobic core of micelles. Poly(lactic-co-glycolic acid) (PLGA) is one of most popular hydrophobic polymers used as a core part for drug encapsulation [10]. PLGA has ester bonds that break down in the body, resulting in sustained drug release. Consequently, many researchers presented natural polymer-PLGA as biodegradable amphiphilic copolymer, e.g., hyaluronan-PLGA, dextran-PLGA, heparin-PLGA and chitosan-PLGA [11,12,13]. Some studies used a multi-benzene ring moiety as the hydrophobic core. When the drug has also many benzene rings, pi-pi interactions between the drug and micelles can affect drug loading and release profiles [14]. The physicochemical properties of amphiphilic polymers for drug-encapsulated micelles determine the factors that influence the drug delivery features in a similar manner, as chemical linkers do in drug-conjugated micelles. In addition, Abraxane is an albumin-bound form of paclitaxel with a mean particle size of approximately 130 nm as an anti-cancer chemotherapy drug that paclitaxel exists in a non-crystalline, amorphous state. It appears to provide greater access of the drug from the bloodstream to the tumor tissue and permits a higher dose of drug with a decreased infusion time [15,16,17].

#### 2.1.2. Bilayer Vesicles

Some lipids and linear block copolymers with a particular hydrophilic/hydrophobic ratio can self-assemble to form vesicles, liposomes and polymersomes. These vesicles are closed bilayers whose core parts comprise the same solvent as that on the surface of the vesicles. Therefore, these vesicles are suitable to deliver water-soluble drugs or biomaterials, including enzymes, antibodies or genes [18,19].

Panchagnula *et al.* reported that polyethylene glycol-coated liposomes encapsulating water-soluble prodrugs were successfully prepared to overcome their physical instability. They used rat plasma in an *in vitro* hydrolysis study and showed that the chemical bonds in the prodrug of paclitaxel were quite stable; the liposomes were also stable, and crystallization of paclitaxel was not observed [20]. In addition, bilayer vesicles can be applied for the co-delivery of hydrophilic and hydrophobic therapeutic agents for cancer treatment. It has been demonstrated that polymersomes prepared by using PEG-PLA block-copolymers could be loaded with hydrophobic DOX in their shell and hydrophilic anti-Bcl-2 siRNA in their core. These drug/gene co-loading polymersomes showed high loading efficiency and excellent stability that originated from the polymeric core-shell structures with tunable void size and shell thickness and significant rigidity compared to liposomes. Co-delivery of drugs/genes using polymersomes inhibited the growth of cancer cells with much lower IC_50_ values, suggesting that this system can successfully reduce the side effects of cancer chemotherapy [21]. Liposome delivery systems offer the potential to enhance the therapeutic index of anticancer drugs, either by increasing the drug concentration in tumor cells. Among them, Myocet and Doxil were the first-approved liposome-based drugs for cancer treatments. Both products have longer circulating half-life in blood as compared with the free drug, but Doxil has a much longer circulation time in blood than Myocet [22,23].

#### 2.1.3. Cross-Linked Nanogels

**Figure 2:**
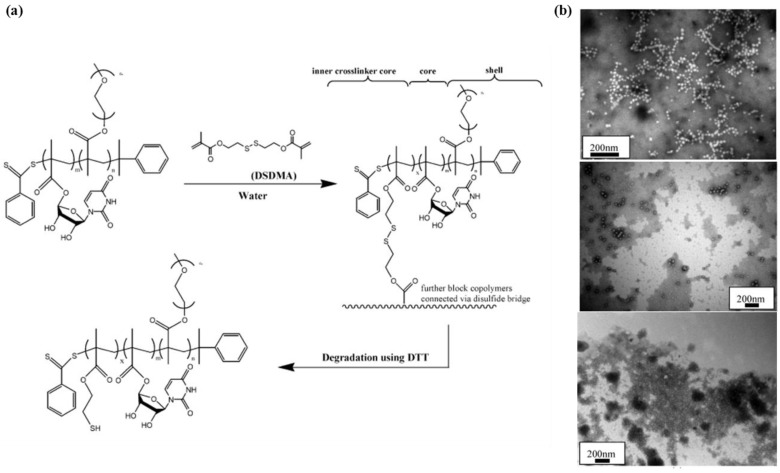
(**a**) Synthesis and Degradation of Core-Cross-Linked Micelles and (**b**) TEM images of (**top**) block copolymer PPEGMEMA_30_-*b*-PMAU_80_, (**middle**) core-cross-linked micelles PPEGMEMA_30_-*b*-PMAU_80_-*b*-PDSDMA_2*45_, (**bottom**) PPEGMEMA30-*b*-PMAU80-*b*-PDSDMA_2*45_ after reduction with DTT, using phosphotungstic acid staining. Reproduced from [26] with permission of [American Chemical Society Publications].

Nanogels are another type of polymeric nanostructure for drug delivery systems that is physically or chemically a cross-linked polymer network in nano-size [24,25]. Nanogels meet the requirements for drug carriers, e.g., high loading capacity, high stability and stimuli-responsive release characteristics for controlled delivery. Drug-loading approaches using nanogels are quiet similar to those of micelles, *i.e.*, conjugation or hydrophobic interactions between the drugs and the nanogel matrix. A main concern in drug delivery using nanogels is to establish cross-linking, which is important to control their drug loading and release characteristics. The degree of cross-linking determines the pore size and swelling property of the nanogel, which is related to drug entrapment and release. Stenzel *et al.* reported that drug carriers prepared by using poly(polyethylene glycol methyl ether methacrylate)-block-poly(5'-*O*-methacryloyluridine) (PPEGMEMA_30_-*b*-PMAU_80_) with a disulfide cross-linking agent, (bis(2-methacryloyloxyethyl)disulfide) showed increased drug loading capacity with increasing cross-linking degree (Figure 2). Disulfide cross-linkers are expected to react with redox species in the cells, e.g., glutathione and dithiothreitol, resulting in environment-specific release profiles [26]. The selection of the cross-linking moiety determines stimuli-responsive release characteristics for controlled delivery; common classification for cross-linkers includes disulfide, reactive amine, click-chemicals for host-guest interaction and photo-reactive chemicals with double bonds [24].

#### 2.1.4. Solid Lipid Nanoparticles

Solid lipid nanoparticles (SLNs) have been utilized as novel approach to drug delivery systems with spherical shape with an average diameter between 10 and 1000 nm. SLNs combine the advantages of lipid emulsion and polymeric nanoparticle systems, which possess a solid lipid core matrix stabilized by surfactants to solubilize liphophilic molecules [27,28,29,30,31,32,33]. They have potential advantages, such as the low toxicity and excellent physical stability, and can stably deliver liphophilic drugs with high loading capacity, while they cause toxic effects because of slow degradation. Therefore, drug release can be controlled released depending on lipid matrix incorporation [28,29]. Choi *et al.* developed doxorubicin-loaded solid lipid nanoparticles (SLN-DOX) using biocompatible compounds and examined their *in vivo* therapeutic effects. Compared with free DOX, SLN-DOX have the potential to serve as a useful therapeutic approach to overcome the chemoresistance of adriamycin-resistant breast cancer [33].

### 2.2. Inorganic Cargoes: Simple Particles, Porous Materials, Hollow Structures

#### 2.2.1. Simple Sphere Nanoparticles

**Figure 3:**
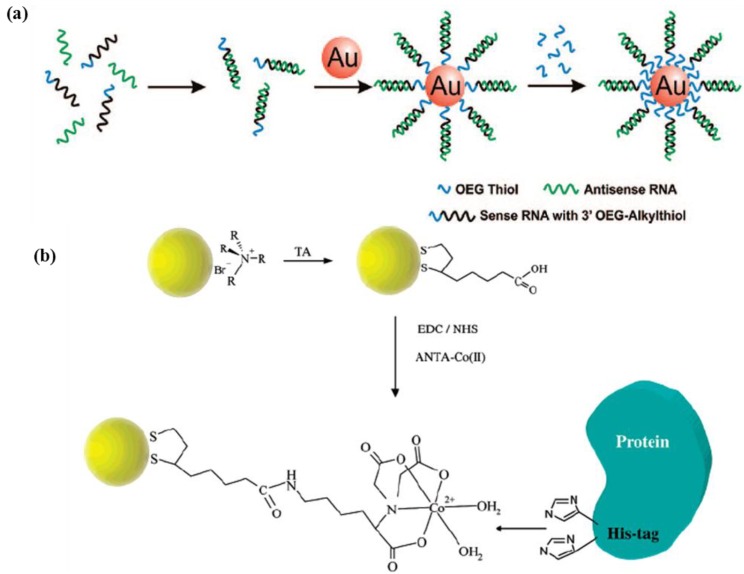
(**a**) Synthesis of polyvalent RNA gold nanoparticle (AuNP) conjugates and (**b**) reaction strategy showing the successive steps for the construction of NTA-terminated nanoparticles for specific immobilization of histidine-tagged proteins. Reproduced from [38,42] with permission of [American Chemical Society Publications].

Inorganic nanoparticles, such as gold nanoparticles (AuNPs), can be attractive cargoes for delivery of drugs, genes and proteins, because of their tunable parameters, e.g., particle size, surface properties and biocompatibility with low toxicity [34,35]. In particular, gold nanoparticles are remarkable drug carriers, because they can provide unique drug release strategies using internal or external stimuli, such as glutathione, pH, heat and light [36,37]. To load drugs on inorganic nanoparticles, they are usually attached to the surface of inorganic nanoparticles by conjugation, charge interaction or hydrophobic interaction [38,39,40]. In particular, attaching the drug to the surface of inorganic nanoparticles is facilitated by thiol groups that are well known for their remarkable interaction with several inorganic nanoparticles, such as gold nanoparticles (Figure 3) [38]. In case of peptide delivery, additional cysteine moieties, which contain thiol groups, are introduced to peptide sequences for coating of the nanoparticles [41]. When charge interaction has to be applied, molecular coating of the surface with opposite charge precedes attachment via thiol groups [39]. Additional histidine moieties are also assumed to be an attractive tag to the metal to load the desirable drug/biomolecules [42].

#### 2.2.2. Porous Nanoparticles

Since mesoporous silica nanoparticles were first reported (MCM-41) for use as drug delivery system in 2001, several remarkable reports have been published using silica-based porous materials, e.g., SBA-15 or MCM-48, as drug carriers [43]. Mesoporous silica nanoparticles have ordered uniform pores for precise control of drug loading and releasing. Their high surface area is also an attractive characteristic for their use as drug carriers, because drugs are usually loaded onto the pore surface by physical adsorption. Drug loading capacity and release profiles can be very different depending on pore diameter, pore topology, surface properties, *etc.* [44]. Andersson *et al.* reported additional factors related to the porosity and kinetic release. They demonstrated the effects of pore connectivity, geometry and matrix degradation in aqueous media, as well [45]. Considering these tunable factors, mesoporous silica nanoparticles were used to deliver a variety of guest molecules, including drugs, such as ibuprofen, doxorubicin (DOX) and cisplatin, therapeutic genes and antibodies [44,45,46,47,48].

Furthermore, the payload molecular stored within these pore domains of mesoporous silica nanoparticles through chemical modification that silica nanoparticles could stimulus-responsively mechanically interlock molecules, mechanized silica nanoparticles. These nanoparticles either change shape or shed off their parts in response to a specific stimulus, such as changes in redox potential, alterations in pH, irradiation with light or oscillating magnetic field, allowing payload molecules to release from the nanopores to a precise location at the appropriate time [49]. Luminescent porous silicon nanoparticles were developed, which are able to carry a drug payload and monitor their accumulation and degradation *in vivo* by intrinsic near-infrared photoluminescence. In addition, these particles could self-destruct into renally cleared components relatively fast, thus it showed low-toxicity [50].

#### 2.2.3. Hollow Nanoparticles

**Figure 4:**
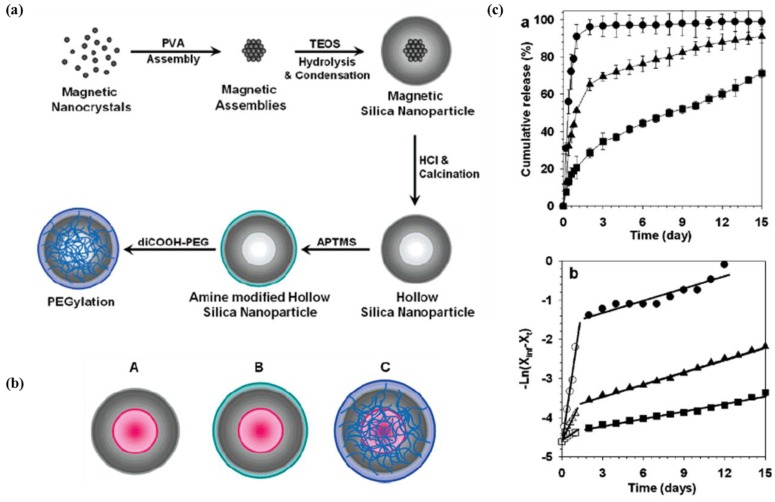
(**a**) Schematic illustration of drug-loaded hollow silica nanoparticles for drug delivery vehicles; (**b**) three types of hollow silica nanoparticles (HSNPs) as drug delivery vehicles: (**A**) HSNPs-OH, (**B**) HSNPs-NH_2_ and (**C**) HSNPs-PEG; (**c**) DOX release profiles and semilogarithmic plot of DOX release of HSNPs (●), HSNPs-NH2 (▲) and HSNPs-PEG (■). Reproduced from [52] with permission of [American Chemical Society Publications].

Hollow inorganic nanoparticles represent a unique structure for drug containers. To produce a cavity in the nanoparticle, removable templates are introduced, e.g., polymeric or rather-soft inorganic nanoparticles. Hollow silica nanoparticles, extensively used as drug carrier, have been reported using various templates, including PS−PVP−PEO block copolymer [51], Fe_3_O_4_ clusters [52], and so forth. After silica coating of the templates, the following steps are required for proper removing of the template: dissolution using apt solvents [53] or calcination [51,53] for organic templates and acidic etching for soft inorganic templates [52]. Haam *et al.* reported high drug loading efficiency and sustained release kinetics of a model drug (DOX) using hollow silica nanoparticles with Fe_3_O_4_ clusters as template (Figure 4) [52]. On the other hand, Xia *et al.* reported silver-template hollow gold nanocages with controllable void size, wall thickness and wall porosity by employing an elaborate process of galvanic replacement, alloying and dealloying. With this hollow gold nanocage as the potential drug carrier, they developed a drug delivery system for controlled release with near-infrared light, which takes advantage of the unique optical property of gold in nanoscale, *i.e.*, surface plasmon resonance [54].

### 2.3. Hierarchical Organic/Inorganic Hybrids

**Figure 5:**
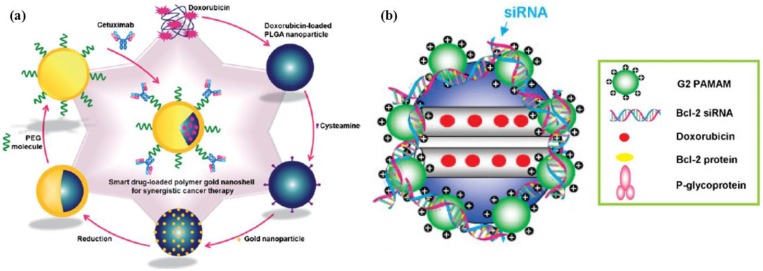
(**a**) Schematic illustration of multifunctional drug-loaded gold nanoshells for synergistic cancer therapy; (**b**) schematic diagram of a co-delivery system based on mesoporous silica nanoparticles (MSNs) to deliver DOX and Bcl-2-targeted siRNA simultaneously. Reproduced from [56,57] with permission of [Willey].

To deliver two or more therapeutic agents, organic/inorganic hybrids can be applied as the drug delivery carrier [55]. Inorganic nanoshells with a polymer core matrix were prepared to deliver therapeutic antibodies and anticancer drugs. In this study, the anticancer drug DOX was incorporated in biodegradable PLGA nanoparticles by applying the nanoemulsion method using poly vinyl alcohol as the amphiphilic polymer (Figure 5a). After gold shell coating on the DOX-loaded PLGA nanoparticles, cetuximab, which is a therapeutic antibody for the epithermal growth factor receptor, was conjugated onto the gold nanoshell surface using functionalized PEG as the cross-linker [56]. In addition, porous silica nanoparticles coated with a cationic polymer were used for co-delivery of an anticancer drug and therapeutic gene. In this report, DOX was adsorbed onto mesoporous silica nanoparticles, as described above. In another study, mesoporous silica nanoparticles were coated with G2 amine-terminated poly(amido amine) *(PAMAM)* dendrimers to load anti-Bcl-2 siRNA as a therapeutic gene by charge-charge interaction (Figure 5b) [57]. The drug loading principles for these organic/inorganic hybrids obey those of each system to which the respective drug belongs. However, the drug release pattern of hybrids could be quiet different to those of a separated system, because the local environment in the hybrid structure could alter the drug. A highly charged G2 amine-terminated PAMAM dendrimer layer can act as a barrier against the diffusion of hydrophobic DOX from the core of mesoporous silica nanoparticles.

## 3. Enhanced Drug Accumulation at Target Sites: Pharmacokinetics and Biodistribution

### 3.1. General Physiological Strategies: Size Modulation for Enhanced Circulation Half-Life, Reticuloendothelial system (RES) Avoidance by Surface Modification (PEG)

Drug delivery carriers remain in the circulation via the reticuloendothelial system (RES), e.g., liver and spleen, depending on their size and surface characteristics. To overcome their drawbacks, various parameters, including size and surface modification, have been studied. Among surface modification strategies, PEG as hydrophilic polymers can increase the half-life due to their high water solubility by hydrogen bonding between their oxygen atoms and water, as well as they can reduce the uptake by the RES through protection against degrading enzymes [58,59]. PEG-modified polymers have been researched for effective drug delivery. Kataoka used PEG-containing block copolymer micelles as drug-delivery carriers that led to the development of dendritic and star-shaped amphiphilic structures, which exhibit enhanced control over architecture, size and surface functionality of micelles compared to linear block copolymers.

### 3.2. Cancer-Specific Physiological Strategies

#### 3.2.1. Passive Targeting by EPR Effects

Tumors can present an increased production of several mediators and enzymes, which altogether enhance the permeability of tumor vessels with respect to those of normal tissues, because of the rapid vascularization. In addition, little or no lymphatic drainage in tumors leads to passive accumulation and retention of nanoparticles with prolonged circulation times in the tumor resulting in an enhanced permeation and retentions (EPR) effect [60,61,62,63,64]. In order to reach the target solid tumor site, the circulation time of drug carriers in the blood should be increased, which can be achieved by incorporating well-characterized macromolecules, e.g., PEG, polyacrylic acid, polyvinyl alcohol, dextran, chitosan and polyethyleneimine. DOX, as an anticancer drug, incorporated into PEG-coated liposomes, is currently in use on clinical conditions and demonstrated high efficacy in EPR-based tumor therapy with low side effects [60]. Haam *et al.* developed PEG-modified drug carriers for reducing toxicity and increasing circulation time [65,66,67]. These carriers effectively delivered the drugs to tumor cells and showed high therapeutic effects.

#### 3.2.2. Active Targeting by Molecular Binding Receptor (Antibody, Targetable Polymer, *etc.*)

**Figure 6:**
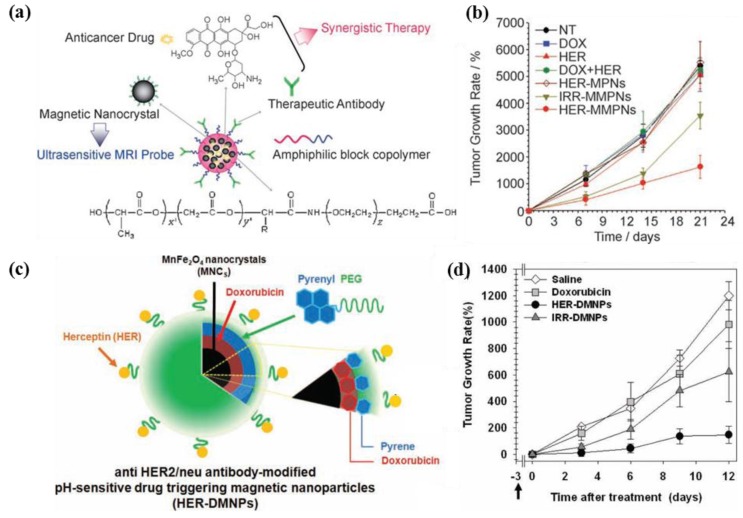
Schematic illustration of (**a**) multifunctional magneto-polymeric nanohybrids (MMPNs) and (**c**) Herceptin-modified pH-sensitive drug-delivering magnetic nanoparticles (HER-DMNPs) for cancer therapy, respectively. Comparative therapeutic efficacy study of MMPNs (**b**) and HER-DMNPs (**d**) in the *in vivo* model. Reproduced from [14,70] with permission of [Willey].

Selectively delivering drugs to target tumors can serve to improve the therapeutic efficiency in cancer treatment, while reducing side effects in normal tissues. Drug delivery carriers that are modified by specific surface markers (targeting moieties) enable their specific recognition by target cells, which facilitates effective delivery to target tumor tissues [68,69,70,71,72,73]. As targeting moieties, antibodies, peptides (arginine-glycine-aspartate; RGD), nucleic acids (aptamers), polysaccharides (hyaluronic acid [HA]), glycoproteins (transferrin) and small molecules (folate) are extensively employed [11,14,65,66,69,70,71,72,73,74,75,76,77,78,79,80,81,82,83,84,85]. For example, Haam *et al.* developed anti-HER2/neu antibody (Herceptin^®^; HER)-modified drug carriers for effective therapy of HER2/neu receptor overexpressing breast cancer [14,67]. This carrier showed synergistic therapeutic effects of DOX and HER as a therapeutic antibody by suppressing the cell growth signals on the HER2/neu receptor, which led to more effective tumor growth inhibition compared to control groups, resulting from target-specific delivery to tumor sites through receptor-mediated endocytosis (Figure 6). Especially, DOX are released form the pyrenyl groups of the polymer in an acidic condition owing to decreased in π–π interactions between DOX and the pyrenyl groups (Figure 6c). From *in vivo* results, these particles were confirmed excellent synergistic therapeutic efficacy (Figure 6b,d). In addition, hyaluronic (HA)-coated drug carriers (HCDs) were successfully synthesized for targeted delivery of DOX to CD44-expressing human breast cancer cells. Because of its solubility in aqueous solutions, HA could strongly interact with the CD44 receptor and demonstrated increased half-life in the body. It has been reported that HCDs have therapeutic potentials in cancer treatment by increasing the tumoricidal efficacy on target cancer cells, while reducing their cytotoxicity to non-targeted cells in order to minimize the side effects [74]. Aptamer-conjugated polyplexes (APs) were developed containing shRNAs against Bcl-xL and DOX in combination cancer therapy. These APs demonstrated synergistic and selective cancer cell death through AP-mediated co-delivery of very small amounts of DOX- and Bcl-xL-specific shRNAs by an intrinsic apoptotic pathway [77]. Recently, Ruoslahti *et al.* reported that tumor-homing peptide (iRGD)-mediated compounds were delivered into the tumor parenchyma that allow significant improvement of the sensitivity of imaging agents and enhance the activity of therapeutic agents [76,77,78,79,80,81,82].

#### 3.2.3. Active Targeting by Magnetic Guidance

Therapeutic delivery by magnetic guidance is an effective strategy for the selective delivery to target sites with drug release control. In this case, drug delivery carriers containing magnetic nanoparticles have been utilized, in which magnetic particles were guided along an externally placed magnet, and thus, drug in company of magnetic nanoparticles could be delivered to the desired site. Therefore, this approach achieved significantly high localization and retention in the target region with low unwanted effects of chemotherapy [64,86,87,88]. Gang *et al.* prepared magnetic polymeric nanoparticles, magnetic PCL nanoparticles, containing an anticancer drug (gemcitabine) to develop a more efficient drug delivery system for cancer therapy [89]. The magnetic PCL nanoparticles showed high therapeutic effects by delivering drugs efficiently to magnetically targeted tumor tissues; in addition, they could be used as magnetic resonance (MR) probes to detect cancer. This system could be applied for targeted therapy with cancer detection.

## 4. Ongoing Advances in Drug Delivery Systems

### 4.1. Stimuli-Responsive Drug Delivery

Stimuli responsive drug delivery systems are investigated for remotely controlled drug release by specific external or internal stimuli, including light [89,90,91,92,93,94], magnetic field [95,96,97,98,99,100,101,102], ultrasound [103,104,105,106,107,108,109], pH [100,101,102,103,104,105,106,107,108,109,110,111,112,113,114] and specific enzymes’ activity [115,116]. These systems allow the drug concentration to be maintained within its therapeutic window to target sites and to release the drug by changing the structures of their components.

#### 4.1.1. Light-responsive Drug Delivery Systems

**Figure 7:**
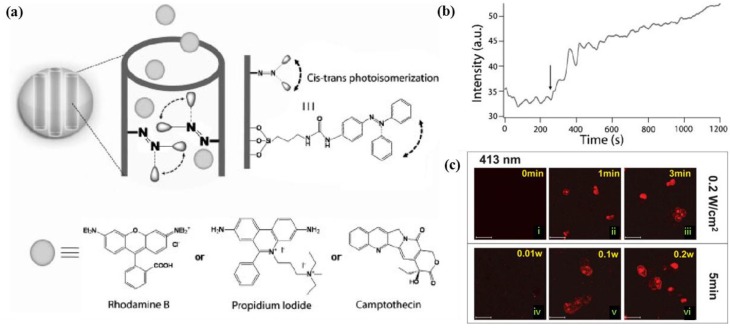
(**a**) Designed pore interiors of the light-activated mesostructured silica (LAMS) nanoparticles functionalized with azobenzene derivatives; (**b**) Time-dependent release of Rhodamine B dye from the LAMS into water. The arrow indicates the time at which the azobenzene activation light was turned on; (**c**) Confocal microscopy images of the photocontrolled staining of PANC-1 cancer cells. Propidium iodide (PI)-loaded LAMS was incubated with the cells for 3 h in the dark. The cells were then exposed to the activation beam for 1 to 5 min. After further incubation in the dark for 10 min, the cells were examined with confocal microscopy (λ_ex_= 337 nm). PANC-1 cancer cells incubated with the PI-loaded LAMS and illuminated for 0 (i), 1 (ii), 3 (iv) or 5 min (vi) under a constant = 0.2 W cm^2^, 413 nm light or with different light intensities (=0.01 (iii) or =0.1 W cm^2^) (v) for 5 min with a 413 nm light. Scale bar = 30 μm. Reproduced from [93] with permission of [Willey].

Light as an external stimulus causes structure and temperature changes in systems, which can be used for the spatiotemporal control of drug release. Drug delivery systems with light-activated materials have been designed using various strategies, e.g., drugs conjugated with nanoparticles via photo-cleavable ligands. After light-irradiation, the drug can be trigger-released by cleaving or activating its linkage [89,90,91,92]. Lu *et al.* developed nanoimpeller-based delivery systems, light-activated meso-structured silica particles containing molecular impellers, could regulated the drug release inside of living cell by remotely controlling both light intensity and the irradiation time (Figure 7) [93].

In addition, drug loaded onto nanoparticles can be released from the polymeric matrix to the outside layer through diffusion by temperature change. This is possible due to the hydrogen bonding among polymer networks that become weak as the temperature increases [94].

Recently, Haam *et al.* developed a novel nanotherapeutic system consisting of a PLGA matrix containing DOX as a chemotherapeutic agent and a gold over-layer on a polymer matrix capable of exhibiting a photothermal effect [56]. Upon light (near-infrared) irradiation, DOX can be abruptly released from the polymer matrix for high cancer cell toxicity, and photothermal therapy can be applied by using the heat from the gold shell. In addition, drugs were conjugated with drug delivery systems via light-activated ligands, which can be used for controlled released using external light irradiation by activating cleavage of its specific ligands, e.g., *o*-nitrobenzyl linkage [90].

#### 4.1.2. Magnetically-Triggered Drug Delivery Systems

**Figure 8:**
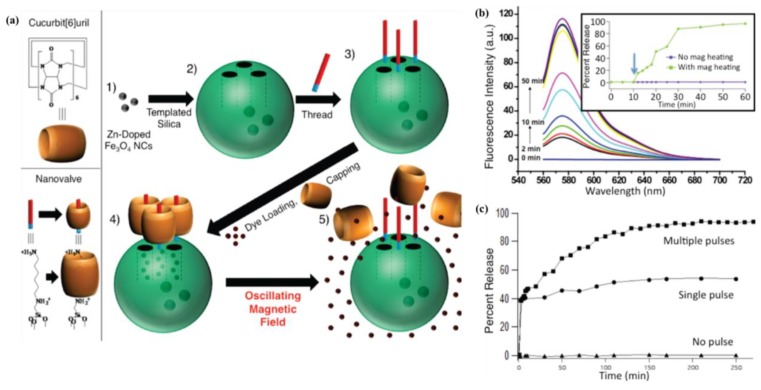
(**a**) Illustration of synthetic route of magnetically activated release system (MARS) nanoparticles. ZnNCs (**1**) are synthetically positioned at the core of the mesoporous silica nanoparticles (**2**). The base of the molecular machine is then attached to the nanoparticle surface (**3**). Drug is loaded into the particle and capped (**4**) to complete the system. Release can be realized using remote heating via the introduction of an oscillating magnetic field (**5**). The particles and machines are not drawn to scale. Cargo is released using magnetic actuation; In (**b**), the MARS nanoparticles were continuously exposed to the magnetic field. The inset shows the data as a release profile; In (**c**), a sample was kept at 0 °C and exposed to pulses of the magnetic field. A single AC magnetic field exposure (●) exhibited ~40% cargo release after an initial 1 min pulse. Multiple pulses performed at 1, 3, 5, 7 and 9 min and then every 20 min for 270 min (■) enabled more dye release until all of the dye diffused out. A baseline (▲) was obtained by monitoring the fluorescence with no pulses. The low temperature of the surrounding solution (0 °C) was maintained in order to observe the effects only from the magnetic field and not from heating of the surrounding solution. Reproduced from [101] with permission of [Willey].

Magnetic nanoparticles produce heat through various energy losses under an external alternating magnetic field because of the transformation of their magnetic energy into heat by the dynamic response of a dipole with their magnetic moments [95,96,97,98]. Hyperthermia by magnetic field causes cancer destruction by activating cell-death signaling. Magnetic nanoparticles are not only magnetically hyperthermic, but they are also drug delivery or actuators capable of controlled drug release [99,100,101,102]. Recently, Cheon *et al.* developed an on-demand drug delivery/release system as a magnetothermally responsive system for highly effective *in vivo* cancer treatment. This system showed a high tumor suppression effect with low drug dosage compared to before (Figure 8) [99,100,101].

#### 4.1.3. Ultrasound-Mediated Drug Delivery Systems

It has been reported that the interaction of ultrasound with nanoparticles could enhance drug delivery in tumors cells, because this affects the properties of tumor vasculature and cell membrane and induces non-thermal effects by nanoparticle oscillation and acoustic streaming. This interaction allows enhancement of drug delivery [103,104,105,106,107,108,109]. Ultrasound-absorbed nanoparticles could control the drug-releasing behavior of nanoparticles and their distribution. In addition, ultrasound has practical advantages in therapeutic usage, because of clinical accessibility, low cost and safety.

#### 4.1.4. pH-Responsive Drug Delivery Systems

Cancer cells produce more lactic acid than normal cells by increased glycolysis and proton-pump activity; the acid is released to extracellular regions, leading to a lower extracellular pH (pH 6.5 to 7.2) than blood and normal tissues (pH 7.4). On the basis of this feature, pH-responsive drug carriers have been actively developed to facilitate specific responses to cancer cells without activation under normal physiological conditions [110,111,112,113,114]. Recently organic/inorganic nanoparticles containing aromatic molecules were investigated, of which the π (pi)–π (pi) interaction was affected by ionization due to pH changes that resulted in drug release [14]. Moreover, novel pH-sensitive nanosphere designed for colon-specific delivery were prepared using polymeric mixtures of poly(lactic-co-glycolic) acid (PLGA) and a pH-sensitive methacrylate copolymer, and this nanosphere showed strongly pH-dependent drug release properties in acidic condition and particulate targeting ability against specific colon cells in inflammatory bowel disease [111].

#### 4.1.5. Enzyme-Responsive Drug Delivery Systems

Recently, drugs conjugated with nanoparticles via peptide linkers enable triggered release by specific enzymatic activation, in, which a specific peptide sequence is hydrolyzed or cleaved in the presence of specific enzymes (cathepsin B, caspase) or protein antigens, e.g., matrix metalloproteinases [115,116].

### 4.2. Theranostic Nanoparticles

**Figure 9:**
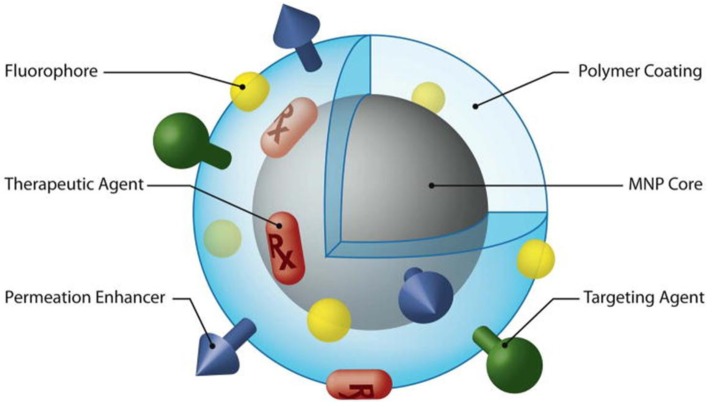
Magnetic nanoparticle (MNP) possessing various ligands to enable multifunctionality from a single nanoparticle platform. Reproduced from [92] with permission of [Elsevier].

Nanoparticles have been developed in combination with myriad payload drugs, imaging agents and targeting moieties, leading to the formulation of theranostic nanoparticles capable of delivering therapy concomitant with diagnosis. Co-localization of the MR image (MRI) detection site and drug release site ensures increased anticancer treatment efficacy; thus, this system allows early and accurate diagnosis, efficient cataloguing of patient groups for personalized cancer therapy and real-time monitoring of disease progress (Figure 9) [117,118,119,120,121,122,123,124,125,126,127,128]. Haam *et al.* developed multifunctional magneto-polymeric nanohybrids composed of magnetic nanocrystals and anticancer drugs encapsulated by an amphiphilic block copolymer. The antibody-modified nanohybrids exhibited ultrasensitive targeted detection by MRI with excellent synergistic effects on tumor growth inhibition (Figure 6a,b) [70]. Brody *et al.* also synthesized polyacrylamide-based hydrogel particles containing a PET imaging probe as the theranostic carrier against lung cancer [128]. These studies demonstrated that this approach could be used as a novel nanodrug delivery system for the simultaneous diagnosis and treatment of various types of cancers.

In particular, the application of these emerging systems in personalized therapy requires the development of stimuli-responsive, activatable nanomaterials that are capable of producing chemical or physical changes *in vivo*. In addition, they need to be able to respond appropriately to subtle *in vivo* changes for the controlled release of the therapeutic content, to obtain information on the metabolism and to be activated at an appropriate time for therapy and signal induction. Therefore, activatable nanomaterial-based therapies generating only localized effects are intrinsically less invasive than conventional therapies, such as surgery and nonspecific drug delivery. They are expected to increase the efficacy of therapeutic agents, while reducing unwanted side effects, such as toxicity. In addition, they could greatly curb the systemic side effect of anticancer drugs by regulating drug release kinetics [11,14,129,130,131,132,133].

Notably, activatable theranostic nanoparticles could be effective in cancer therapy in that the loss of drug is minimized until the target is known to exist in the patient, as well as in monitoring and verifying that the target has been reached and that the therapy is working. Recently, pH-activatable theranostic agents were developed, in which organic-soluble anticancer drugs can be trapped along with magnetic nanoparticles inside an amphiphilic polymer that delivers anticancer drugs to cancer cells and effectively labels tumors for MRI [14]. The extracellular pH of tumors is more acidic (pH 5–6) than blood and normal tissues (pH 7.4), because of the unique microenvironment of tumors, *i.e.*, lactic acid is produced by tumors and released to extracellular regions. This fact has been actively exploited to develop drug carriers that can specifically respond to cancer cells with low pH values while remaining inactive at normal physiological conditions (Figure 6c,d) [134]. The drug release profiles of these pH-responsive drug carriers can be understood as a function of the pH value. Thus, the additional pH-responsive function of theranostic nanocarriers could give rise to a potent, multi-tasking, all-in-one system for cancer therapy. In this method, the drug delivery site coincides with the MRI detection site. Furthermore, upon reaching the target site, the drug release profile, which can be used to estimate the amount and release duration of the delivered drug, can be closely monitored to assess treatment efficacy. These properties could greatly aid in the decision-making process for patient-specific drug administration strategies, moving closer to the full implementation of effective cancer therapy, which would generate innovations and play a critical in nanomedicine [14].

## 5. Conclusion

In the past decades, the use of nanotechnology for drug delivery systems has grown exponentially. On the basis of considerable advances in the fabrication of drug nanocarriers with organic and/or inorganic architecture, worldwide pre-clinical researches have been underway with the current understanding of cancer, which undoubtedly expanded more than ever before. This review focused on drug delivery systems in cancer treatment using nanotechnology, providing an overview of the physicochemical principles of fine delivery systems targeted for cancer and cancer environments, whose pathophysiological characteristics are the strategic gateway for efficient nanoscale therapy. Ongoing developments have further expanded the boundary of this paradigm in medicine, such as the concept of “theranosis”, a system that can be used to perform diagnosis and therapy simultaneously. Although there have been toxicity and safety issues, we believe that we will benefit from the new knowledge of molecular events in cancer gathered by nanoscale drug delivery systems. With the continued discovery of new materials, the establishment of improved designs and considerate efforts for sophisticated optimization, we predict that a “cancer-overcoming era” will emerge.
